# Stanniocalicin 2 Suppresses Breast Cancer Cell Migration and Invasion via the PKC/Claudin-1-Mediated Signaling

**DOI:** 10.1371/journal.pone.0122179

**Published:** 2015-04-01

**Authors:** Jing Hou, Ziliang Wang, Han Xu, Lina Yang, Xiaoli Yu, Zhaozhi Yang, Yun Deng, Jiao Meng, Yan Feng, Xiaomao Guo, Gong Yang

**Affiliations:** 1 Department of Radiation Oncology, Fudan University Shanghai Cancer Center; Department of Oncology, Shanghai Medical College, Fudan University, Shanghai, China; 2 Cancer Institute, Fudan University Shanghai Cancer Center; Department of Oncology, Shanghai Medical College, Fudan University, Shanghai, China; 3 Breast surgery, Fudan University Shanghai Cancer Center; Department of Oncology, Shanghai Medical College, Fudan University, Shanghai, China; 4 Central Laboratory, The Fifth People’s Hospital of Shanghai, Fudan University, Shanghai, China; Sun Yat-sen University Medical School, CHINA

## Abstract

Stanniocalcin (STC), a glycoprotein hormone, is expressed in a wide variety of tissues to regulate Ca^2+^ and PO4^-^ homeostasis. STC2, a member of STC family, has been reported to be associated with tumor development. In this study, we investigated whether the expression of STC2 is associated with migration and invasion of breast cancer cells. We found that breast cancer cell line 231 HM transfected with STC2 shRNA displayed high motility, fibroblast morphology, and enhanced cell migration and invasion. Introduction of STC2 in 231 cells reduced cell migration and invasion. In response to irradiation, silencing of STC2 in 231 HM cells reduced apoptosis, whereas overexpression of STC2 in 231 cells promoted apoptosis, compared with in control cells. Mechanistic study showed that STC2 negatively regulated PKC to control the expression of Claudin-1, which subsequently induced the expressions of EMT-related factors including ZEB1, ZO-1, Slug, Twist, and MMP9. Suppression of PKC activity by using a PKC inhibitor (Go 6983) restored the normal motility of STC2-silenced cells. Furthermore, *in vivo* animal assay showed that STC2 inhibited tumorigenesis and metastasis of breast cancer cells. Collectively, these results indicate that STC2 may inhibit EMT at least partially through the PKC/Claudin-1-mediated signaling in human breast cancer cells. Thus, STC2 may be exploited as a biomarker for metastasis and targeted therapy in human breast cancer.

## Introduction

Stanniocalcin constitutes a small family of secreted homodimeric glycoproteins first found in the corpuscles of Stannius and has been implicated functional in the physiology of Ca^2+^ and PO4^-^ homeostasis, metabolism, reproduction, stress response and development [1–5]. The STC family contains two members, STC1 and STC2. STC2 consists of 302 amino acids and exhibits ~60% homology to STC1 [6]. The expression of STC2 has been identified to be involved in a variety of cancers including renal, breast, and ovarian cancers [7–12]. Numerous studies have reported that the STC2 gene can be epigenetically modified and the expression of STC2 may be regulated by stimulation of hypoxia and/or endoplasmic reticulum (ER) stress in human cancers [4,9]. Gene profiling studies showed that STC2 was significantly elevated in a specific subset of breast cancer[13]. However, the prognostic value of STC2 in breast cancer is still controversial. Iwao et al. reported that the expression of STC2 was associated with better prognosis of breast cancer and that loss of the STC2 expression indicated poor prognosis [14]. High expression of STC2 mRNA was associated with good outcome in certain breast cancer patients [15,16]. Thus, the function of STC2 in breast cancer is still elusive.

Epithelial-mesenchymal transition (EMT) is a process that cancer cells may lose their epithelial properties to acquire a mesenchymal phenotype and become motile and invasive [17–19]. The EMT process is orchestrated by a number of factors, including ZEB1, Slug, Snail, Twist and Vimentin [20–25]. Law et al. reported that STC2 could promote EMT in hypoxic ovarian cancer cells [26]. However, little is known about the correlation between STC2 and EMT in breast cancer cells. In the present study, by silencing or overexpression of STC2 in aggressive breast cancer cell lines, we found that STC2 might regulate EMT through the activation of Protein Kinase C (PKC).

## Materials and Methods

### Cell Lines and Cell Culture

Human breast cancer cell lines MCF-7, ZR-7530, MDA-MB-231(231) (expressing low STC2) and lentiviral packaging cell line (293T cell) were purchased from American Type Culture Collection (Manassas, VA). MDA-MB-231 HM (231 HM) cells (expressing high STC2) were established by Breast Cancer Institute of Fudan University Shanghai Cancer Center [27]. All cell lines were maintained in DMEM medium, supplemented with 10% fetal bovine serum, penicillin (100 units/mL), and streptomycin (100 μg/mL). All cell cultures were incubated at 37°C in 5% CO2 atmosphere.

### Chemicals

Go 6983, a PKC inhibitor, was purchased from Selleck and dissolved in DMSO. The final DMSO concentration of the solution used throughout the study did not exceed 0.1%. Cells were grown to 70–80% confluence on plates and treated with 1 μM of Go 6983 for 12 h. Then the cells were digested with trypsin and used in the following experiments.

### Cloning of STC2 cDNA and transfection

RNA isolated from SKOV3 cells was used for reverse transcription with PrimeScript 1st Strand cDNA Synthesis Kit (TaKaRa, Japan) according to the protocols provided. The in-frame coding region of STC2 was PCR-amplified and inserted into the *EcoRI* and *BamHI* sites of the pCDH-CMV-MCS-EF1-Puro vector. The forward primer is 5’-TATGAATTCGCCACCATGTGTGCCGAGCGGCT-3’; reverse primer is 5’-ATGCGGATCCTCACCTCCGGATATCAGAAT-3’. The sequence of the STC2 insert was verified by DNA sequencing. Restriction enzymes were purchased from New England Biolabs, T4 DNA ligase from promega. Primers were synthesized by Sangon Biotech (Shanghai, China). The shRNA of STC2 were purchased from Genechem (Shanghai, China). Lentiviruses expressing STC2 cDNA or STC2 shRNA and their corresponding empty vectors were produced by transfection of plasmids into 293T cells and used to infect target cells (231 and HM cells) by using a method described before [28]. Cells were selected with puromycin (1.5μg/mL) for 10–14 days.

### Cell Proliferation assay

Cells were detached by trypsinization and washed twice with PBS. 2×10^3^ cells per well were incubated in 96-well culture plates (Corning Inc., Corning, NY) in 100 μl medium. The cells were incubated for 12 h to allow for attachment, after which a 0-time point measurement was determined. After culturing for 1, 2, 3, 4, 5 days, the supernatant was removed, and cell growth was detected using Cell Counting Kit-8(CCK-8) (Dojindo Laboratories, Kumamoto, Japan) according to the manufacturer’s instructions. Absorbance at 450 nm was measured using a microplate reader. All proliferation assays were performed independently at least 3 times.

### Migration and Invasion assays

For cell migration assay, we used a high throughput screening multi-well insert 24-well two-chamber plate (BD Biosciences, San Jose, CA), with an 8-μm (pore size) polycarbonate filter between chambers. 4×10^4^ of 231 STC2 or HM STC2i cells and their corresponding controls (231 Vector or HM Scr, respectively) were added in each upper chamber and allowed to migrate at 37°C for 12 hours toward a lower reservoir containing medium plus 2.5% fetal bovine serum. For cell invasion assay, cells were seeded into Matrigel-coated inserts and were incubated in the media with 2.5% serum for 24 h. Non-invading cells on the upper side of the insert membrane were removed with cotton swabs. The invaded cells were fixed with ice-cold methanol for 30min, and stained with 0.1% crystal violet for 15 min, then photographed and counted at ×200 magnification under a microscope. The assay was repeated three times with duplication each time.

### Scratch-wound motility assay

To detect migration speed by scratch assay, cells were incubated in 6-well plate over-night to yield monolayer confluence for scratch assay. Scratches were made using a pipette tip (200μl) and photographed immediately (time 0) and at 24 h, 36h. The distance migrated by the cell monolayer to close the scratch area during this time period was measured. Experiments were carried out in triplicate and repeated at least three times.

### Colony formation assay

To test colony formation, cells were plated (3–5×10^2^ cells in 6-wellplates) in triplicate in DMEM supplemented with 10% FBS. After10 day’s incubation, the cells were washed and fixed with ice-cold methanol for 30min, and stained with 0.1% crystal violet for 15 min, then photographed and counted under microscope.

### Examination of Cell Apoptosis

To detect apoptosis, 1 × 10^5^ cells were stained with Annexin V and propidium iodide, according to the protocol in Annexin V-FITC Apoptosis Detection Kit (BD Biosciences, NJ, USA), and subject to analysis with Flow Cytometry (FC500 MPL, Beckman Coulter, CA, USA). The percentage of apoptotic cells was calculated in terms of peaks (M2) appearing in histograms, representing an early apoptotic population (Annexin V+/PI−) among the total cells analyzed. The experiment was done in duplicate and repeated three times.

### Real-time PCR

Total RNA was isolated from 2 × 10^6^ 231 STC2 or HM STC2i cells, and their control cells, by using Trizol reagent (Invitrogen, Carlsbad, CA). cDNAs generated by reverse transcription were used for real-time PCR analysis with the ExScript RT-PCR kit (TaKaRa, Japan). qPCR primers for STC2 are as follows: 5’-GGGTGTGGCGTGTTTGAATG-3’ (sense) and 5’-CTTGAGGTAGCATTCCCGCT-3’ (antisense). qPCR primers for GAPDH are as follows: 5’- ACCCAGAAGACTGTGGATGG-3’ (sense) and 5’-TCTAGACGGCAGGTCAGGTC-3’ (antisense). All amplifications and detections were carried out in the LightCycler 480 system (Roche, Basel, Switzerland) using the LightCycler 480 SYBR Green I Master (Roche, Basel, Switzerland) and the following program: 95°C for 10s, one cycle, and 95°C for 5s and 62°C for 31s, 40 cycles, followed by a 30-min melting curve collection, which was used to verify the primer dimers. Statistical analyses were performed using the 2-ΔΔCT relative quantification method.

### Western blotting

To analyze protein expression in cells, cell lysates were prepared at 75% of confluence by using 500 μL of radioimmunoprecipitation assay buffer (25 mM Tris—HCl at pH 7.6, 150 mM NaCl, 1% Nonidet P-40, 1% sodium deoxycholate, and 0.1% sodium dodecyl sulfate) in 10-cm culture dish, with a 20-minute incubation on ice. Protein concentrations of the lysates were measured with a Bio-Rad protein assay kit (Hercules, CA). Immunoblot analyses were performed as previously described[29]. STC2 and β-actin antibodies were obtained from Santa Cruz Biotechnology. pPKC, Claudin-1, MMP9, Slug, Twist, Vimentin, ZEB1,ZO-1 were purchased from Cell Signaling Technology. The secondary antibodies were F(ab)2 fragment of donkey anti-mouse immunoglobulin (product NA931) or of donkey anti-rabbit immunoglobulin (product NA9340) linked to horseradish peroxidase from Amersham Biosciences (Little Chalfont, Buckinghamshire, UK). Immunoblot reagents were from an electrochemiluminescence kit (Amersham Biosciences).

### Xenograft Tumors in nude mice

231 cells or 231 HM cells stably transfected with STC2 cDNA or shRNA, or control vector or scrambled shRNA by lentiviral infection were used for animal assays. To generate tumor growth in vivo, 4 × 10^6^ cells of each cell line were subcutaneously injected into 4- to 6-week-old BALB/c athymic nude mice (Department of Laboratory Animal, Fudan University). The animal experiments were approved by the Institutional Animal Care and Use Committee of Fudan University and performed following Institutional Guidelines and Protocols. Each cell line was injected into the fat pads of 12 mice for a total of 12 injections. The longest diameter “a” and the shortest diameter “b” of tumors were measured and the tumor volume was calculated with the use of the following formula: tumor volume (in mm^3^) = a × b^2^ × 0.52, where 0.52 is a constant to calculate the volume of an ellipsoid. When a tumor reached 1.5 cm in diameter, all mice were sacrificed. Tumors, lungs, and lymph nodes were excised and subjected to routine HE staining examination by investigators who were blinded to the tumor status.

## Results

### STC2 maintains breast cancer cell morphology

**Fig 1:**
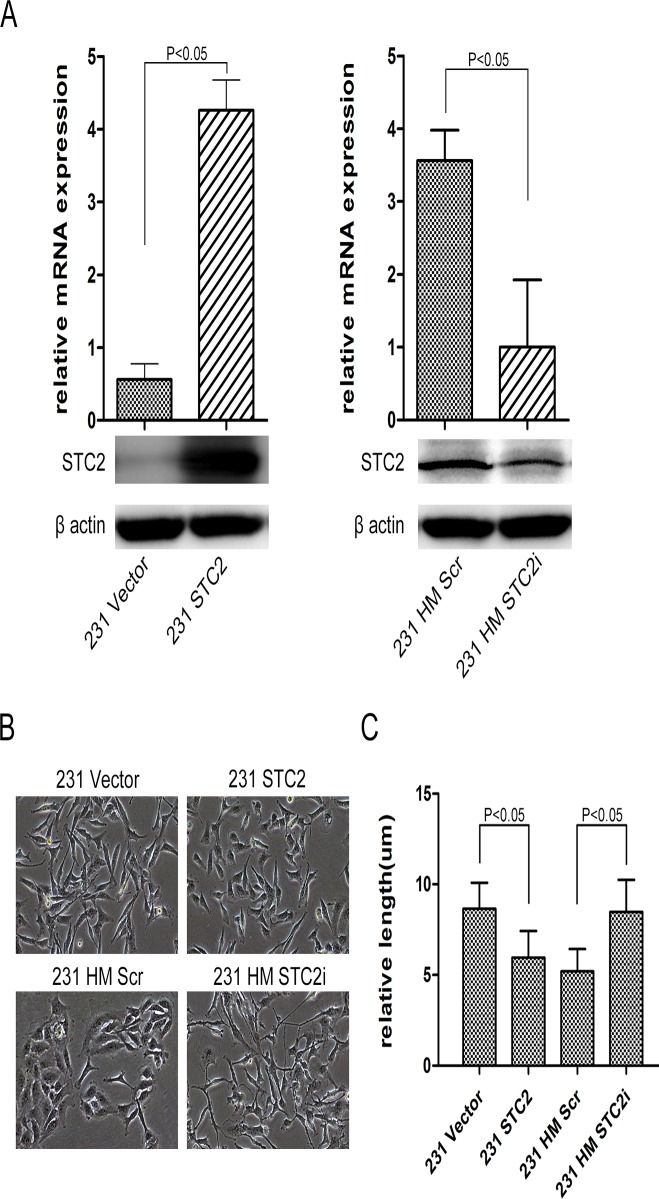
STC2 maintains breast cancer cell morphology. **A**, Analyses of STC2 by Western blotting and realtime-PCR in constructed cells (P < 0.05). Error bars = 95% confidence intervals (CIs). **B**, Cell morphology. Ectopic overexpression of STC2 in 231 cells changed the cell spindle phenotype, and displayed as frizzle phenotype. 231 HM cells losing STC2 expression displayed an elongated mesenchymal-like morphology with scattered distribution. **C**, Measurement of the relative length of the cells according to **Fig 1. B** (P < 0.05). Error bars = 95% CIs.

To investigate the function of STC2 in breast cancer cells, we chose two breast cancer cell lines, MDA-MB-231 for overexpression of STC2, and MDA-MB-231 HM for silencing of STC2, because we had confirmed by q-PCR that MDA-MB-231(231) cells had low STC2 expression, whereas MDA-MB-231 HM (231 HM) cells had moderate expression of STC2 (data not shown). To establish these cell lines, lentiviruses carrying STC2 cDNA or shRNA against STC2 were generated and used to infect 231 cells or 231 HM cells, respectively (corresponding control cells were infected with empty vector or scrambled shRNA viruses). Establishment of cell lines was confirmed by q-PCR and Western blotting (Fig. 1A). By using phase contrast microscope, 231 cells were viewed as spindle phenotype, introduction of STC2 in 231 cells changed the morphology to frizzle phenotype. However, knockdown of STC2 in 231 HM cells changed the morphology from cobblestone to elongated mesenchymal-like phenotype (Fig. 1B). To present the results in a quantitative way, the relative length of attached cells was measured as shown in Fig. 1C. 231 STC2 cells became shorter than corresponding control cells. On the contrary, 231 HM STC2i cells appeared longer than 231 HM Scr cells.

### STC2 has little effect on cell proliferation

**Fig 2:**
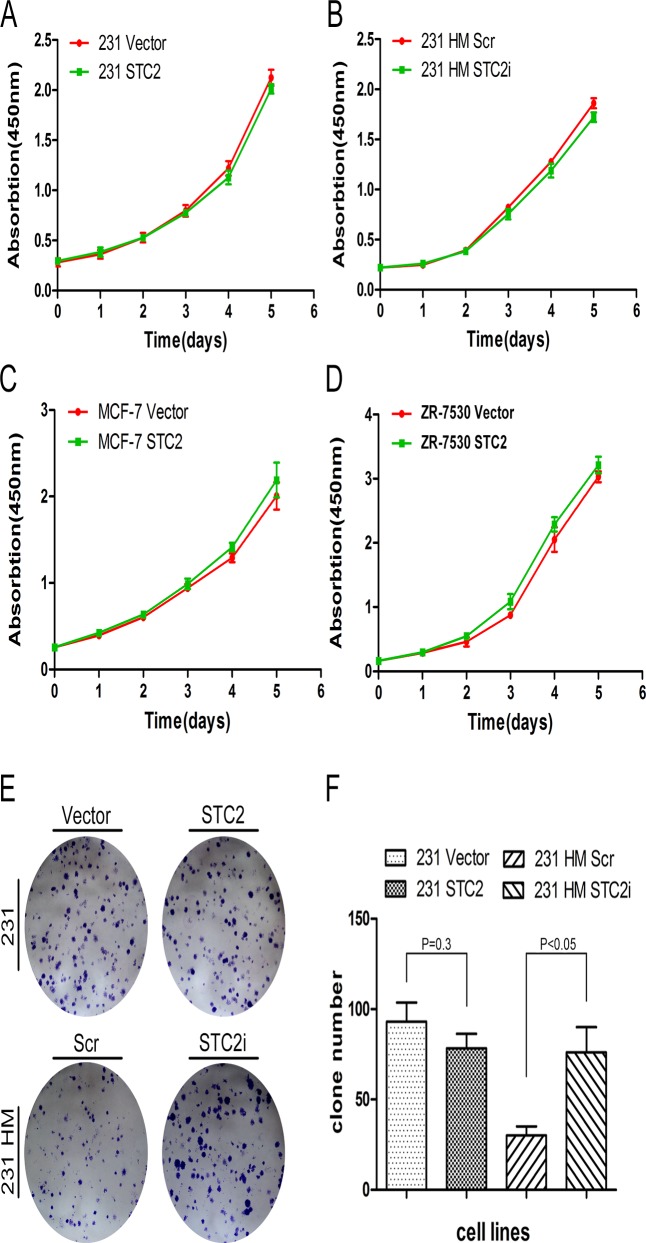
STC2 has little effect on cell proliferation. **A-D**, Detection of cell proliferation by CCK8 (P < 0.05). Error bars = 95% CIs. **E-F**, Colony formation assay. **E**, Representative images of 231 STC2 cells and 231 HM STC2i cells and their corresponding controls, and **F**, Quantitative analysis of the number of clones (P < 0.05). Error bars = 95% CIs.

Some studies [9,11,26] reported that STC2 enhances cell proliferation, however, other studies [5,6,12,30,31] showed that STC2 inhibits cell proliferation. Thus, the effect of STC2 on cell proliferation is elusive [32]. In this study, we found that no different growth was observed between 231 STC2 or 231 HM STC2i and their corresponding control cells. This notion was further confirmed by overexpressing STC2 in two more breast cancer cell lines MCF-7 and ZR-75-30 (Fig. 2A-D). Furthermore, we also found that the number of colonies formed by 231 STC2 cells was almost similar to that formed by 231 Vector cells. But the number of colonies formed by 231 HM STC2i cells was nearly two times more than that formed by control cells (Fig. 2E-F).

### STC2 suppresses migration and invasion of breast cancer cells

**Fig 3:**
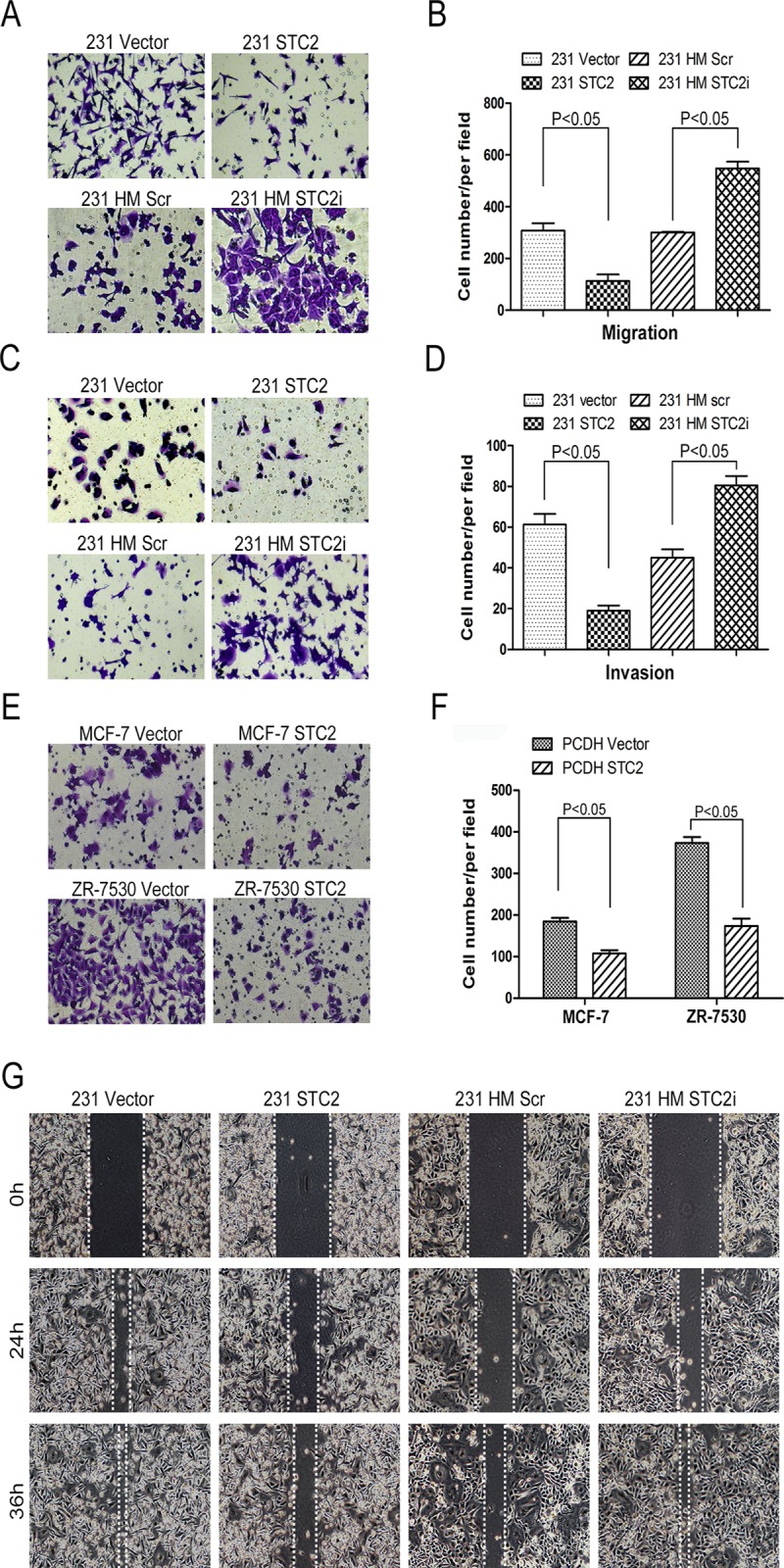
STC2 influences migration and invasion of breast cancer cells. **A-B**, Migration assays. **A**, Representative images of migrated cells. **B**, Quantitative analysis of the number of migrated cells. Five images were taken randomly for each experiment, and each experiment was repeated three times (P < 0.05). Error bars = 95% CIs. **C-D**, The invasion assay (P < 0.05). Error bars = 95% CIs. **E-F**, STC2 influences migration of breast cancer cells MCF-7 and ZR-75-30. **E**, Representative images of migrated cells. **G**, Quantitative analysis of the number of migrated cells (P < 0.05). Error bars = 95% CIs. **G**, Scratch-wound motility assay, cells were incubated in 6-well plate over-night to yield monolayer confluence. Scratches were made using a pipette tip (200μl) and photographed immediately (time 0) and at 24 h, 36h.

Migration and invasion are hallmarks for cancer cells to metastasize in different organs. To investigate the role of STC2 in migration and invasion of breast cancer cells, we performed migration assays using a high throughput screening multi-well insert 24-well two-chamber plates. We found that fewer 231 STC2 cells migrated through the membrane in the migration chamber than did 231 vector cells (Fig. 3A-B), which was also confirmed in two more breast cancer cell lines MCF-7 and ZR-75-30 by overexpressing STC2 (Fig. 3E-F), but more 231 HM STC2i cells migrated through the membrane than did 231 HM Scr cells (Fig. 3A-B). Similar results were seen for invasion assay (Fig. 3C-D). We also detected migration speed by scratch assay. We observed that the migration speed of 231 STC2 cells was reduced after 24h and 36h culture, compared with control cells. However, the migration speed of 231 HM STC2i cells was increased compared with control cells at the same time points (Fig. 3G).

### STC2 promotes cell apoptosis

**Fig 4:**
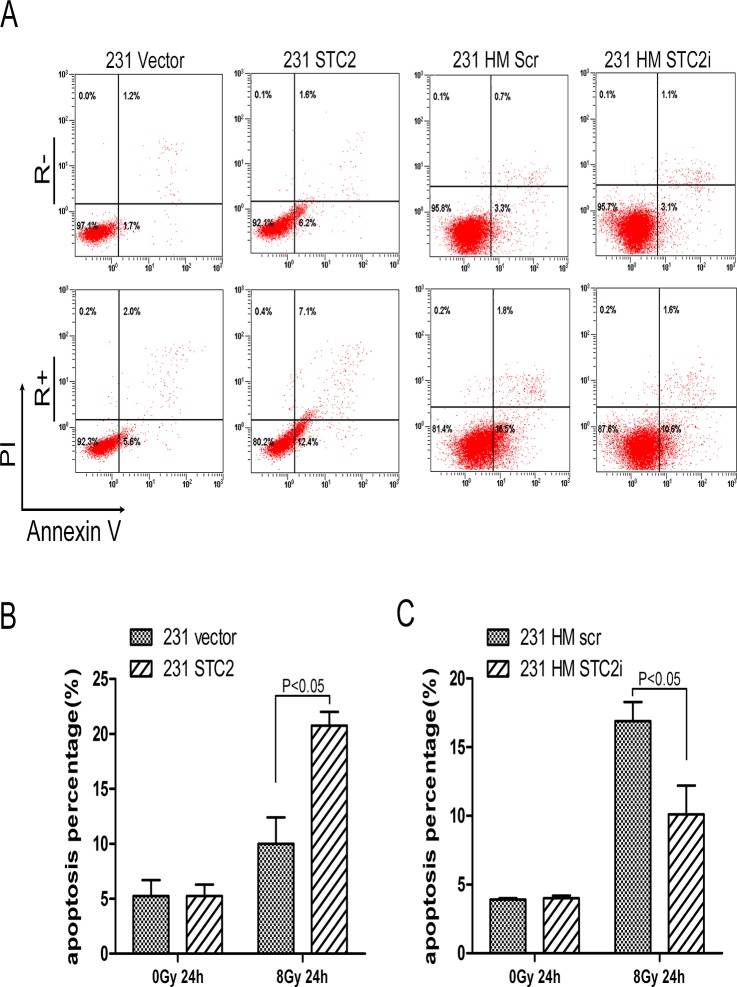
STC2 promotes cell apoptosis. Cells were treated with a dose of 8Gy X-rays, apoptosis was then measured by flow cytometry in 231 STC2 cells or 231 HM STC2i cells and their corresponding control cells. **A-B**, Representative images of apoptosis assay. **C**, The percentage of apoptotic cells (P < 0.05). Error bars = 95% CIs. R+: with Radiation Treatment, R-: without Radiation Treatment.

Many evidence have proved that cells with invasive property are anti-apoptotic. To extend the function of STC2 in breast cancer, we investigated the influence of STC2 expression on apoptosis in breast cancer cells by use of Annexin V fluorescence apoptosis assay. Because the baseline apoptosis of the cancer cells is low, cells were treated with 8Gy X-ray irradiation and the apoptotic percentage was analyzed by FCM. We found that the apoptotic percentage of 231 STC2 cells was higher than that of control cells, while silencing of STC2 in 231 HM cells resulted in less apoptotic than in control cells (Fig. 4).

### STC2 regulates EMT through PKC/Claudin-1 pathway

**Fig 5:**
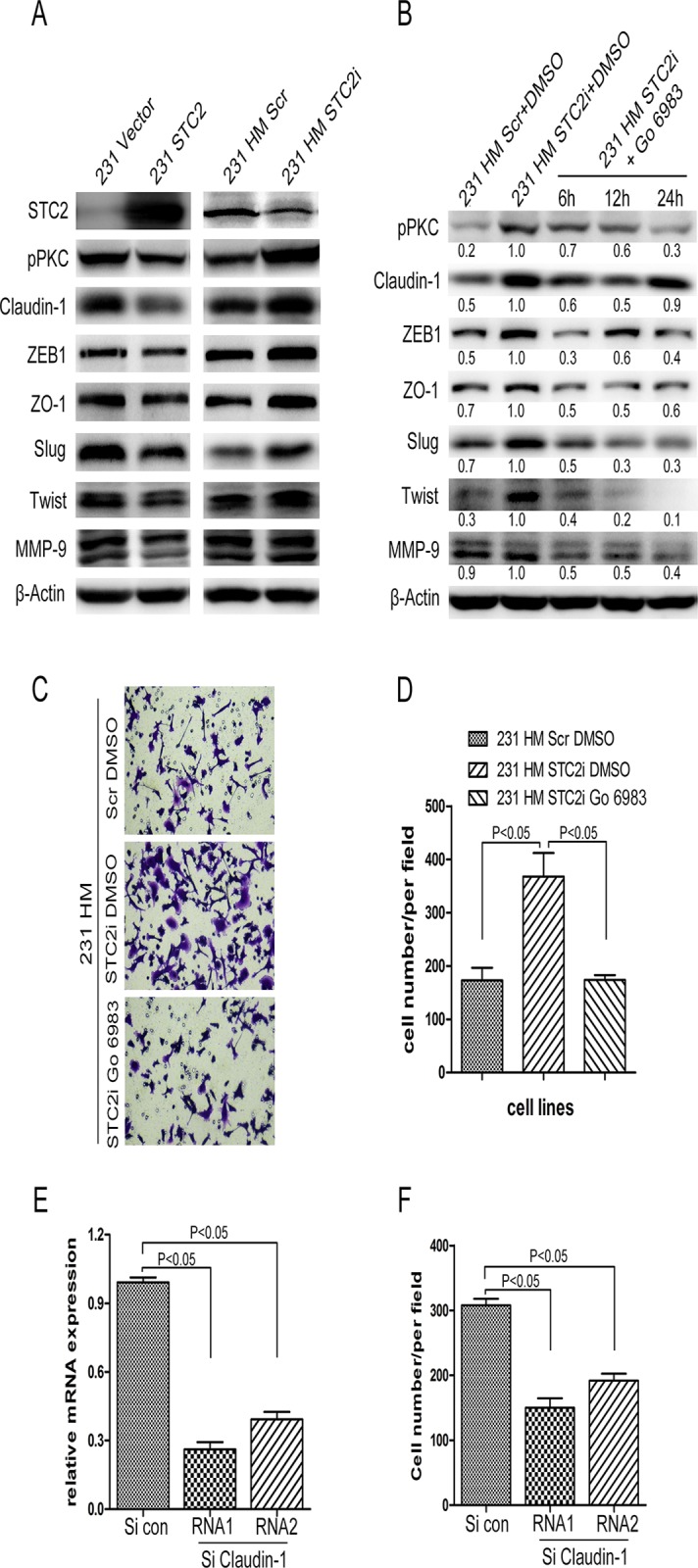
STC2 regulates EMT program through PKC/Claudin-1 pathway. **A**, Immunoblot analysis of molecules associated with PKC/Claudin-1 signal pathway and EMT program. pPKC, Claudin-1, ZEB1, ZO-1, Slug, Twist, MMP9 were measured by Western blotting with the corresponding antibodies. β-Actin was used as the loading control. **B**, Immunoblot analysis and quantitative analysis of pPKC, Claudin-1, ZEB1, ZO-1, Slug, Twist, MMP9 after treatment with 1μM Go 6983 in 231 HM STC2i cells at different time point. **C-D**, Migration assay tested with 231 HM STC2i cells after treatment with PKC inhibitor Go 6983. 231 HM Scr cells and 231 HM STC2i cells were treated with DMSO or Go 6983. 12 hours later, the cells were seeded to perform migration assay (P < 0.05). Error bars = 95% CIs. **E-F**, The migration assay after using siRNAs against Claudin-1, E, Analyses of Claudin-1 by realtime-PCR. F, Quantitative analysis of the number of migrated cells (P < 0.05). Error bars = 95% CIs.

Because STC2 is a glycoprotein hormone that regulates Ca^2+^ homeostasis in tissue, and Ca^2+^ as a second massager can activate Protein Kinase C (PKC). PKC is an important protein regulating many properties of cancer cells, such as proliferation, migration, invasion, apoptosis, and carcinogenesis through activation of many downstream proteins [33–35]. Leotlela et al. found that PKC regulates melanoma cell invasion via PKC/Claudin-1/MMP2 pathway [36]. Thus, we hypothesized that STC2 might exert its function through PKC/Claudin-1/MMPs signaling in breast canbcer cells. To test this hypothesis, we detected the expression of pPKC, Claudin-1, and MMP9 by Western blotting. As shown in Fig. 5A, the protein level of pPKC was reduced in 231 STC2 cells, compared with in control cells. On the contrary, pPKC was increased in HM STC2i cells, compared with in 231 HM Scr cells. The expression of Claudin-1 was altered when STC2 expression level was changed. Intriguingly, we found that the expression of MMP9 was also reduced in 231 STC2 cells, and was up-regulated in 231 HM STC2i cells.

Claudin-1 was reported to induce epithelial-mesenchymal transition (EMT)[37]. Thus, we also detected EMT-associated factors by Western blotting, the results showed that the expressions of ZEB1, ZO-1, Slug, and Twist were reduced in 231 STC2 cells, but were increased in 231 HM STC2i cells (Fig. 5A). These observations suggest that STC2 may regulate cell motility through PKC/Claudin-1 pathway. To further confirm if the PKC/Claudin-1 axis regulates cell migration and invasion, we treated 231 HM STC2i cells with a PKC inhibitor-Go 6983 to, and then detected the expressions of pPKC and Claudin-1 at different times. We found that the protein levels of pPKC and Claudin-1 were reduced after cells were treated at 6, 12, and 24 hours (Fig. 5B). Treatment of cells with Go 6983 also reduced the expressions of ZEB1, ZO-1, Slug, Twist, and MMP9 (Fig. 5B). We also performed migration assay to test if the PKC inhibitor could inhibit the migration of 231 HM STC2i cells. The results showed that more 231 HM STC2i cells migrated through the membrane than 231 HM Scr cells after treated with diluent (DMSO). Treatment with Go 6983 reduced the number of migrated 231 HM STC2i cells (Fig. 5C-D). Furthermore, we used two siRNAs against Claudin-1 to silence the expression of Claudin-1 in 231 HM STC2i cells, and then performed the migration assay. The results showed that knockdown of Claudin-1 impaired the migratory ability of 231 HM STC2i cells (Fig. 5E-F). As PI3K/AKT and Ras/Erk pathways usually play crucial roles in cell motility, we detected proteins related to these pathways by Western blotting, but the expression levels of the major proteins in PI3K/Akt or Ras/Erk pathways were not influenced by up- or down-regulation of STC2 (S1 Fig.). In all, the above results indicate that the increased cell motility of 231 HM STC2i cells may mainly depend on the activity of PKC.

### Knockdown of STC2 enhances tumorigenicity and breast cancer metastasis

**Fig 6:**
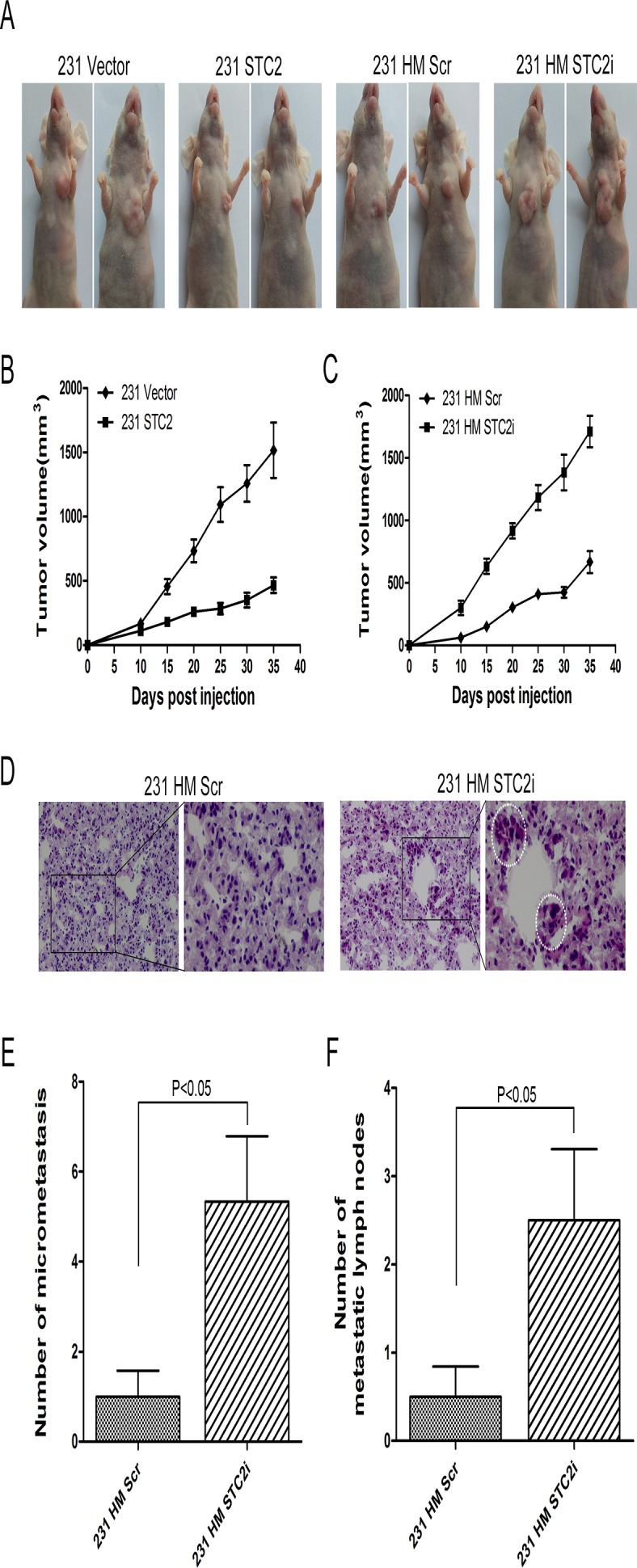
Knockdown of STC2 enhances tumorigenicity and metastasis. **A**, In vivo tumor growth examined by animal assay. **B-C**, Fat pad tumor growth from mice injected with 231 STC2 cells and 231 HM STC2i cells and their corresponding control cells (P < 0.05). Error bars = 95% CIs. **D**, HE staining of the lung tissues from mice injected with 231 HM STC2i cells and their corresponding control cells. **E**, Quantitative analysis of the number of micrometastasis in lungs (P < 0.05). Error bars = 95% CIs. **F**, Quantitative analysis of the number of metastatic lymph nodes (P < 0.05). Error bars = 95% CIs.

To test the effect of STC2 on tumor growth, 231 STC2 cells or 231 HM STC2i cells and their corresponding control cells were injected into the fat pads of nude mice. As shown in Fig. 6A-C, the mice injected with cells expressing STC2 cDNA displayed small tumor volumes, while those injected with cells expressing STC2 shRNA developed big tumor volumes, compared with mice injected with control cells. We also found that the number of micrometastasis in lungs from the mice injected with 231 HM STC2i cells was more than that from control mice (Fig. 6D-E). Furthermore, 67% of mice from 231 HM STC2i cells group developed axillary lymph node metastasis, compared to 33% of the mice from control group. We also found that average number of metastatic lymph nodes of 231 HM STC2i cells group was more than that from control group (Fig. 6F). These results suggest that STC2 may suppress breast tumor growth and metastasis, which is consistent with the in vitro data that STC2 inhibits breast cancer cell invasion and migration.

## Discussion

A number of studies have reported that STC2 plays an important role in human carcinogenesis. However, different correlations between STC2 expression and cancer progression have been reported. Functional studies have not been carried out to fully reveal the impact of STC2 in cancer cells. In our study, using STC2 cDNA overexpressing 231 cells and STC2 shRNA overexpressing 231 HM cells, we identified that STC2 was a negative regulator of cell migration and invasion. Loss of STC2 expression in 231 HM cells transformed cells from the cobblestone phenotype to a more aggressive mesenchymal-like phenotype. Intriguingly, STC2 had little impact on cell proliferation of breast cancer cells in our study. This was inconsistent with some other reports. Raulic et al [30] reported that overexpression of STC2 resulted in a significant impairment of cell growth in breast cancer cells. Volland et al [6] also reported that STC2 reduced the proliferation of neuroblastoma cells in vitro, but increased the invasive potential, which might facilitate early metastasis. In addition, Gagliardi et al [5] demonstrated that the constitutive expression of human STC2 in transgenic mice, resulted in a significant reduction of intramembranous and endochondral bone development, suggesting that STC2 can act as a potent growth inhibitor in vivo. However, Ito et al [4] reported that overexpression of STC2 resulted in the selective protection of HeLa cells against endoplasmic reticulum stress induced cell death. Another study performed by Law et al [9] showed that overexpression of STC2 in SKOV3 cells significantly stimulated a faster proliferation rate under hypoxia. These controversial observations may be due to that STC2 responds differently to stress stimuli in different settings through different signaling pathways.

The function of STC2 in tumor migration and invasion is also controversial. Law et al [26] considered that cells transfected with STC2 showed significantly higher invasive potential than control cells. This conclusion was supported by the study conducted by Volland et al [6], showing that the STC2-transfected neuroblastoma cells had higher invasive potential. In our study, however, we found that STC2 was a negative regulator of cell migration and invasion. Overexpression of STC2 weakened the capacity of cell migration and invasion. On the contrary, silencing of STC2 promoted the motility of 231 HM cells. These results were also supported by the xenograft animal assay showing that STC2 suppressed tumor growth and inhibited tumor metastasis of breast cancer cells. Our observations were similar to those done by Raulic et al [30], showing that loss of STC2 was positively correlated with a more aggressive breast cancer phenotype. We hypothesized that loss of STC2 can initiate the EMT program. Therefore, we detected the EMT-related markers by Western blotting. We found that the expressions of Slug, Twist, ZEB1, Claudin-1, and ZO-1 were down-regulated in 231 STC2 cells, but were up-regulated in STC2-silenced 231 HM cells. Furthermore, 231 STC2 cells displayed more apoptotic, while 231 HM STC2i cells were more anti-apoptotic after radiation. This observation was consistent with the phenomenon that the EMT-like cells possesses more anti-apoptotic properties. These data suggest that STC2 can regulate EMT process, which is closely associated with metastasis.

It is well known that STC2 is implicated in the physiology of Ca^2+^ homeostasis. Since Ca^2+^ is a second messenger which can activate many downstream targets including PKC. PKC is a well-known protein regulating many properties of cancer. We postulated that PKC might serve as a bridge between STC2 and cell migration and invasion. The results of Western blotting showed that the pPKC level was increased in 231 HM STC2i cells, but was decreased in 231 STC2 cells compared with control cells. Studies [36,38] have reported that PKC promotes melanoma cell motility through regulation of Claudin-1, a member of integral membrane proteins associated with tight junctions and the maintenance of cellular polarity[39]. Recent studies have also provided evidence that Claudin-1 is aberrantly expressed in diverse types of human cancers including hepatocellular carcinomas (HCCs), and that Claudin-1 could induce EMT to enhance cell migration and invasion[37]. Therefore we also detected the expression of Claudin-1 in our study. The same tendency of the Claudin-1 expression was seen compared with pPKC. In Leotlela and coworkers’ report, Claudin-1 regulates cell motility dominantly via MMP2, not MMP9 [36]. Interestingly, MMP9 was also increased when the expression of Claudin-1 was increased in this study. The limitation of the present study is that don’t know if STC2 regulates the PKC activity in a direct or indirect way. Since STC2 acts as a secretive glycoprotein hormone and binds to its putative receptor to regulate calcium homeostasis [40], the phosphorylation or activation of PKC may depend on the activation of the upstream inner-membrane kinases closely associated with the STC receptor, through which to further activate Claudin-1 according to our study and others [36,41]. To identify the upstream kinases of PKC, however, further investigation is needed.

Based on these observations, we conclude that STC2 suppresses EMT to hinder cell migration and invasion via the PKC/Claudin-1-mediated signaling, which may be useful for breast cancer diagnosis and treatment.

## Supporting Information

S1 Fig Analysis of the expression levels of proteins in PI3K/Akt or Ras/Erk pathways. (TIF)
