# Supplementary figures and images for: Stanniocalicin 2 Suppresses Breast Cancer Cell Migration and Invasion via the PKC/Claudin-1-Mediated Signaling
Source: PLoS One. 2015 Apr 1;10(4):e0122179. doi: 10.1371/journal.pone.0122179 (PMC4382185; doi:10.1371/journal.pone.0122179)

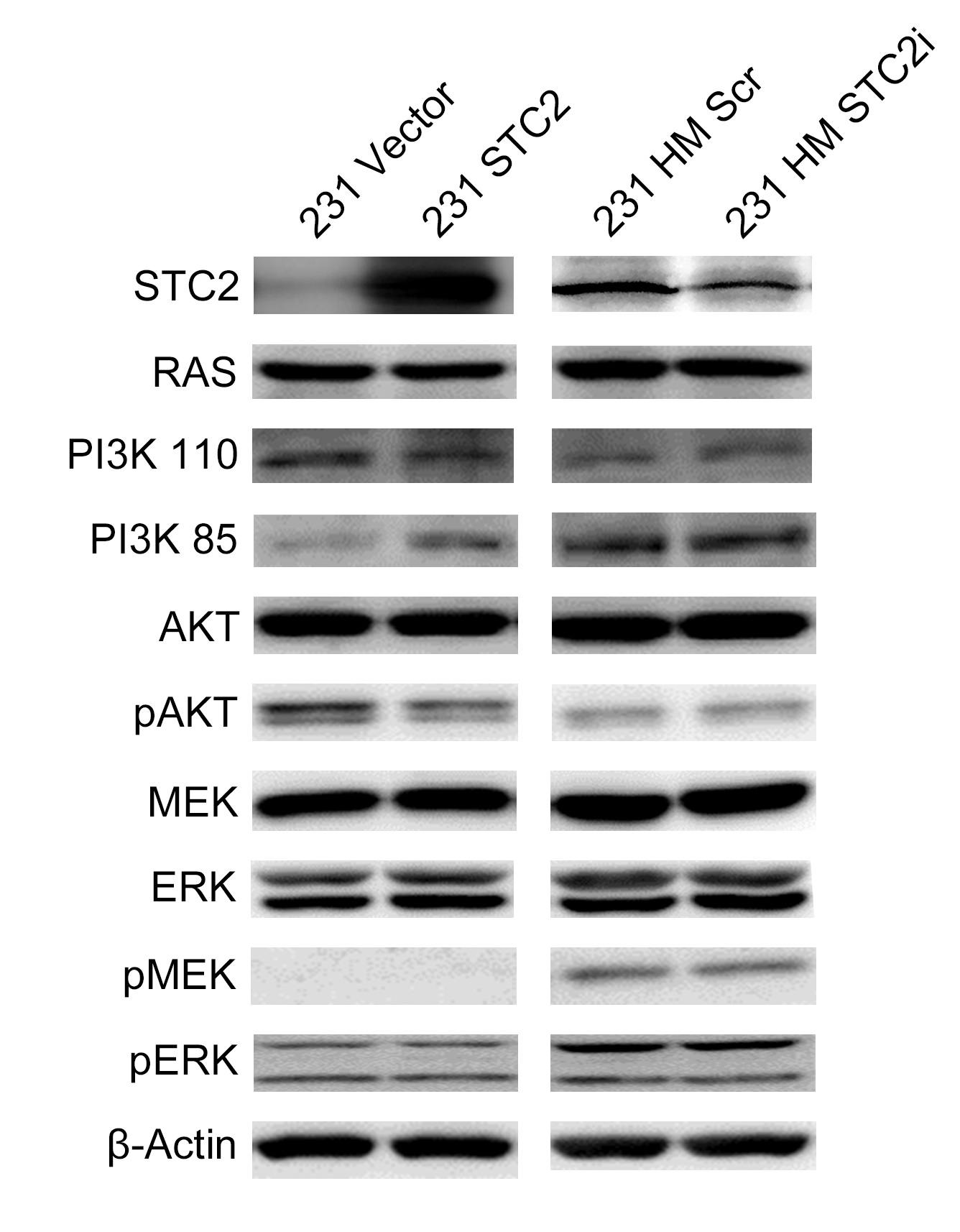

Supplement: S1 Fig — (TIF) [file pone.0122179.s001.tif]
